# Ten Reasons Why People With Down Syndrome are Protected From the Development of Most Solid Tumors -A Review

**DOI:** 10.3389/fgene.2021.749480

**Published:** 2021-11-05

**Authors:** Marta Pilar Osuna-Marco, Mónica López-Barahona, Blanca López-Ibor, Águeda Mercedes Tejera

**Affiliations:** ^1^ Biology of Ageing Group, Faculty of Experimental Sciences, Universidad Francisco de Vitoria, Madrid, Spain; ^2^ Pediatric Oncology and Hematology Unit, HM Hospitals, Madrid, Spain; ^3^ Centro Estudios Biosanitarios, Madrid, Spain

**Keywords:** down syndrome, cancer, metabolism, microRNA, trisomy 21, tumor suppressor genes

## Abstract

People with Down syndrome have unique characteristics as a result of the presence of an extra chromosome 21. Regarding cancer, they present a unique pattern of tumors, which has not been fully explained to date. Globally, people with Down syndrome have a similar lifetime risk of developing cancer compared to the general population. However, they have a very increased risk of developing certain tumors (e.g., acute leukemia, germ cell tumors, testicular tumors and retinoblastoma) and, on the contrary, there are some other tumors which appear only exceptionally in this syndrome (e.g., breast cancer, prostate cancer, medulloblastoma, neuroblastoma and Wilms tumor). Various hypotheses have been developed to explain this situation. The genetic imbalance secondary to the presence of an extra chromosome 21 has molecular consequences at several levels, not only in chromosome 21 but also throughout the genome. In this review, we discuss the different proposed mechanisms that protect individuals with trisomy 21 from developing solid tumors: genetic dosage effect, tumor suppressor genes overexpression, disturbed metabolism, impaired neurogenesis and angiogenesis, increased apoptosis, immune system dysregulation, epigenetic aberrations and the effect of different microRNAs, among others. More research into the molecular pathways involved in this unique pattern of malignancies is still needed.

## Introduction

As Jérôme Lejeune discovered back in 1959 (Lejeune et al., 1959), Down syndrome (DS) is caused by the presence of an extra chromosome 21 (HSA21). In the vast majority of cases it is caused by a complete trisomy, most often due to an alteration in maternal meiosis. Other less frequent causes are mosaicism or unbalanced Robertsonian translocations (mainly between chromosomes 14, 15, 21, or 22) (Hasle et al., 2016; Martel-Billard et al., 2016; DownCiclopedia, 2019; Antonarakis et al., 2020). DS, also known as trisomy 21, is the most frequent cause of intellectual disability caused by a genetic aberration. There are about 5 million people with DS worldwide (Satgé and Rickert, 2009).

People with DS have numerous specific characteristics as a result of the presence of an extra HSA21, which is the smallest human autosome (Hattori et al., 2000; Bénard et al., 2005). It contains 738 genes, of which 233 are coding genes and 182 are pseudogenes (Vega, 2021). The coding genes encode for transcription factors, kinases, ion channels, cell adhesion molecules and proteins involved in cell cycle regulation, DNA or RNA processing and/or modification, among others (Hattori et al., 2000; Florez, 2021).

Murine models have often been used in an attempt to better understand the intrinsic characteristics of DS. Most of the genetic information of HSA21 is represented in the mouse on chromosomes 10, 16 and 17. The most widely used DS murine model is the Ts65Dn mouse, which has a partial trisomy of murine chromosomes 16 and 17, and thus contains a trisomy of 104 genes that are homologous to those in the HSA21 (Aboudafir, 2017; Yu et al., 2020).

It is well known that they have intellectual disability, neurological deficits of variable degree, early aging, neurodegeneration, frequent infections and immune system dysfunction (Antonarakis et al., 2020). Regarding cancer, people with DS should theoretically have an increased risk of developing cancer secondary to the presence of high chromosomal instability, increased DNA damage, defective DNA repair system, high levels of free radicals, mitochondrial dysfunction, constitutional excess of certain oncogenes and an altered immune system (Nižetić and Groet, 2012). In accordance with this idea, other aneuploidies (8, 9, 13, or 18 trisomies, Klinefelter syndrome and Turner syndrome, among others) have an increased risk of developing cancer (Satgé and Bénard, 2008; Nižetić and Groet, 2012). Nonetheless, people with Down syndrome are at similar or slightly lower overall risk compared to the general population (Hasle et al., 2016). In addition, the specific spectrum of tumors they develop is especially remarkable and is scarcely explained so far. On the one hand, they are at high risk of developing both acute lymphoblastic and myeloblastic leukemia (especially megakaryoblastic acute leukemia, of which they have 500 times higher risk), intra- and extracranial germinal tumors, testicular tumors and retinoblastoma. Otherwise, they have a dramatic decrease in the incidence of the vast majority of solid tumors. Among the solid tumors with a very low incidence in people with DS, there are some (e.g., breast cancer, prostate cancer, medulloblastoma, neuroblastoma and Wilms Tumor) that develop only exceptionally (Satgé et al., 1998; Hasle, 2001; Hasle et al., 2016; Ayed et al., 2012; Nižetić and Groet, 2012; Satgé et al., 2013) (Table 1). Numerous epidemiological studies highlight this uniqueness. For example, the odds ratio of developing breast cancer among women with DS is estimated at 0.04 (Yang et al., 2002); no cases of prostate cancer were found in two epidemiological studies that included 1928 (Hasle et al., 2016) and 1888 (Patja et al., 2006) men with DS respectively; and at present only five cases of people with DS diagnosed with neuroblastoma (Satgé and Bénard, 2008), six with Wilms Tumor (Spreafico et al., 2007; Meazza et al., 2020) and two with medulloblastoma (Benesch et al., 2009; Mangum et al., 2016) have been reported.

**TABLE 1:** Reported risks of malignant neoplasms in DS.

Very increased risk	Acute leukemia (Hasle et al., 2000; Boker et al., 2001; Hill et al., 2003; Goldacre, 2004; Patja et al., 2006; Rethoré et al., 2020), both lymphoblastic (Hasle et al., 2000; Boker et al., 2001; Hill et al., 2003; Goldacre, 2004) and myeloblastic (Hasle et al., 2000; Boker et al., 2001; Hill et al., 2003; Goldacre, 2004)
	Testicular cancer (Hasle et al., 2000; Hill et al., 2003; Goldacre, 2004; Patja et al., 2006; Rethoré et al., 2020)
Similar/slightly increased risk	Retinoblastoma (Hasle et al., 2000; Rethoré et al., 2020)
	Intracranial germ cell tumors (Rethoré et al.,^2020^)
	Extracranial germ cell tumors (Rethoré et al.,^2020^)
	Penile cancer (Hill et al.,^2003^)
	Sarcoma of the bone and soft tissue (Rethoré et al.,^2020^)
	Bone tumors (Patja et al.,^2006^)
Reduced risk	Tumors in the CNS (Hasle et al., 2000; Hill et al., 2003; Patja et al.,^2006^; Rethoré et al., 2020)
	Glial tissue tumors in childhood (Rethoré et al., 2020)
	Cancer of the oral cavity (Hasle et al., 2000; Rethoré et al., 2020)
	Upper airway cancer (Patja et al.,^2006^; Rethoré et al., 2020)
	Bronchus cancer (Patja et al.,^2006^; Jørgensen et al., 2019; Rethoré et al., 2020)
	Lung cancer (Hasle et al., 2000; Hill et al., 2003; Patja et al.,^2006^; Jørgensen et al., 2019)
	Uterine cervix cancer (Patja et al.,^2006^; Rethoré et al., 2020)
	Thyroid tumors (Patja et al.,^2006^; Rethoré et al., 2020)
	Skin cancer (Hasle et al., 2000; Patja et al.,^2006^; Jørgensen et al., 2019)
	Head and Neck cancer (Patja et al.,^2006^)
Exceptional (very reduced risk)	Neuroblastoma (Boker et al., 2001; Rethoré et al., 2020)
	Medulloblastoma (Rethoré et al.,^2020^)
	Nephroblastoma or Wilms tumor (Rethoré et al.,^2020^)
	Breast cancer (Hasle et al., 2000; Boker et al., 2001; Hill et al., 2003; Jørgensen et al., 2019; Rethoré et al., 2020)
	Prostate cancer (Patja et al.,^2006^; Rethoré et al., 2020)
Controversial	Lymphoma (Hodgkin and Non-Hodgkin) (Rethoré et al., 2020)
	Increased (Goldacre, 2004)
	Similar (Boker et al., 2001; Hill et al., 2003; Patja et al.,^2006^)
	Reduced (Hasle et al., 2000; Hill et al., 2003)
	Digestive track cancer (Rethoré et al., 2020)
	Increased: overall (Hill et al.,^2003^), colon cancer (Hill et al., 2003; Goldacre, 2004; Patja et al.,^2006^), gallbladder cancer (Patja et al.,^2006^), liver tumors (Hill et al., 2003; Patja et al.,^2006^), gastric tumors (Boker et al., 2001; Patja et al.,^2006^)
	Similar: gastric tumors (Hasle et al.,^2000^), colon cancer (Hasle et al.,^2000^), pancreatic tumors (Patja et al.,^2006^)
	Reduced: overall (Hasle et al.,^2000^), rectal cancer (Patja et al.,^2006^)
	Renal cancer
	Similar (Hasle et al.,^2000^)
	Reduced (Patja et al.,^2006^)
	Unknown (Rethoré et al.,^2020^)
	Bladder
	Increased (Hasle et al.,^2000^)
	Decreased (Patja et al.,^2006^)
	Female genital tract tumors
	Uterine cervix cancer: reduced risk (Patja et al.,^2006^; Rethoré et al., 2020)
	Uterine tumors: similar/slightly increased (Hasle et al., 2000; Rethoré et al., 2020); reduced (Patja et al.,^2006^)
	Ovarian tumors: similar/slightly increased (Hasle et al., 2000; Rethoré et al., 2020); similar (Boker et al.,^2001^); reduced (Patja et al.,^2006^)

In spite of all this, few molecular studies have been developed to answer this enigma. The presence of an extra HSA21 produces an overexpression of many of the genes in this region. This imbalance towards the rest of the genes could explain some of the characteristics of DS (Bénard et al., 2005; Antonarakis et al., 2020). However, this overexpression is neither linear (some genes on HSA21 are overexpressed more than others) nor stable (some genes are transiently overexpressed to a greater or lesser extent depending on the tissue and developmental timing) (Sultan et al., 2007; Spellman et al., 2013; Antonarakis et al., 2020; Yu et al., 2020; Florez, 2021). Of these genes, some might have a positive, negative, or neutral effect on tumor development.

It is worth remembering that studies based on gene and messenger RNA (mRNA) expression levels have limitations, since increased gene expression does not always correlate with increased protein expression. Indeed, an increase in gene expression is sometimes accompanied by a decrease in protein function (Spellman et al., 2013; Florez, 2021).

Identifying the underlying mechanisms that, due to this specific situation of genetic imbalance, protect these individuals from developing tumors of such varied lineage may be fundamental to understanding tumor pathophysiology and, potentially, to identifying key genes in tumorigenesis. This will also enable us to progress in the development of effective therapies both for people with DS and for the general population affected by cancer, and to improve the quality of life for people with DS.

## Molecular Mechanisms that Protect People With Down Syndrome From the Development of Most Solid Tumors

**FIGURE 1:**
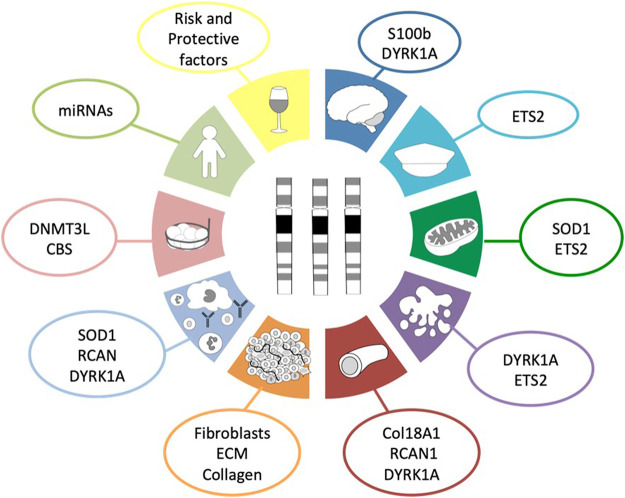
Hallmarks of cancer protection in DS. This diagram summarizes the ten proposed molecular mechanisms and the genes involved in those mechanisms that protect people with Down syndrome from the development of most solid tumors as a consequence of the three copies of chromosome 21. Dark blue corresponds to the role of neural development; turquoise, to the presence of tumor suppressor genes; green, to the disturbed metabolism; purple, to the increased cellular apoptosis and defective DNA repair system; red, to angiogenesis; orange, to the tumor stroma; light blue, to the impaired immune system response; light red, to epigenetics; light green, to global effects outside chromosome 21; and yellow, to non-genetic factors.

Based on scientific evidence and after a thorough bibliographic search, we suggest up to ten reasons that collectively protect people with DS from developing most solid tumors (Figure 1). Along the following pages we are going to review these ten reasons and the possible molecular mechanisms involved in such protection.

### Role of Neural Development

**FIGURE 2:**
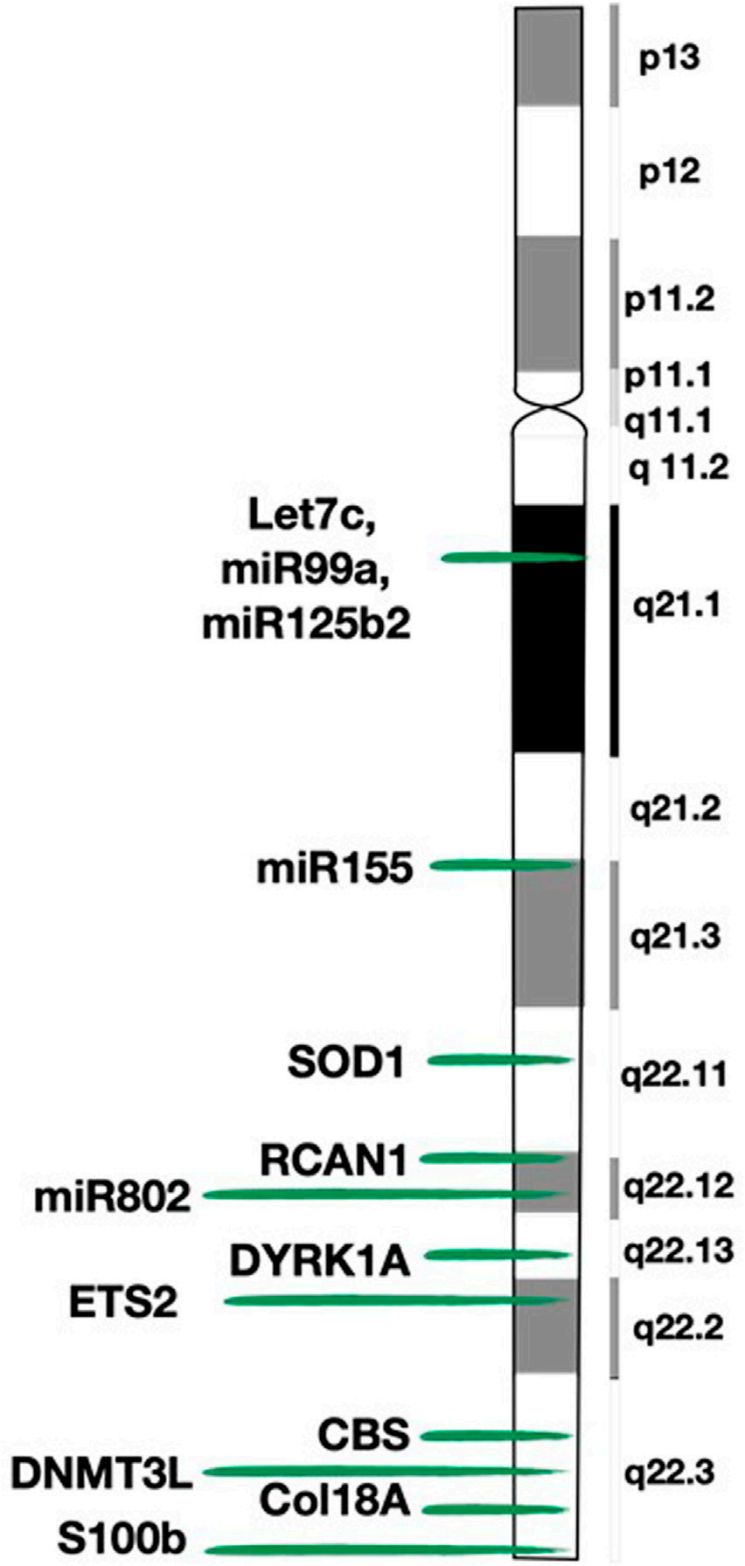
Map of chromosome 21 (HSA21) and location of the genes mentioned in the review. This figure shows the detailed structure of chromosome 21 and the exact location of the genes described in the paper.

It has been observed that people with DS have an underdevelopment of the brain (being smaller and less heavy), of the peripheral nervous tissues, of the cerebellum (with a reduction of the granular cell layer volume) and of the adrenal glands (Satgé and Bénard, 2008; Satgé et al., 2008; Vaillant and Monard, 2009; Wiseman et al., 2009). Moreover, they have an abnormal neural cell population (fewer neurons, reduced neuron size and lower density of synapse network, among others) and a normal glial cell population. It has been demonstrated that transchromosomic tumors (with an extra copy of HSA21) had a three-fold lower mean percentage of neuroectodermal tissue, and the main conclusion drawn from this study is that the supernumerary HSA21 inhibits the differentiation of pluripotent embryonic stem cells (Mensah et al., 2007; Satgé and Bénard, 2008). These results could explain why some type of tumors develop with a standard incidence (e.g., gliomas) but, conversely, others are very rare (e.g., tumors of neural origin) (Satgé and Bénard, 2008). Some authors even propose that the glial population exerts a protective effect on the neural population thus avoiding the development of neural tumors (Satgé and Bénard, 2008). The most relevant genes for neural development present on HSA21 are S100beta and DYRK1A (Figure 2).

The S100B (S100 Calcium Binding Protein Beta) gene (21q22.3) encodes a calcium-binding protein that is secreted by glial cells, and induces neural cell differentiation. *In vitro*, it produces differentiation, apoptosis and growth inhibition in neuroblastoma cell lines (Satgé et al., 2003; Satgé et al., 2008). It is overexpressed in people with DS, Alzheimer disease, brain damage and various tumors (Satgé and Bénard, 2008; Donato et al., 2009). This overexpression in DS can be, on the one hand, beneficial because of its protector effect against neuroblastoma development, but on the other hand, detrimental by favoring neurodegeneration (Satgé et al., 1998). Regarding neuroblastoma, S100B is present at high concentrations in well-differentiated neuroblastomas and with a very low or absent expression in the undifferentiated ones (Satgé et al., 1998). For all this, its overexpression is thought to be responsible, at least to some extent, for the absence of neuroblastoma in children with DS (Hasle et al., 2000; Hasle, 2001; Satgé et al., 2003; Satgé et al., 2008).

The DYRK1A (dual-specificity tyrosine-phosphorylation regulated kinase 1A) gene (21q22.13) is expressed ubiquitously and codes for a protein kinase involved in proliferation and differentiation of neuronal progenitor cells (Abbassi et al., 2015). It has more than twenty direct targets that connect it to multiple pathways including, among others, the cell cycle, apoptosis and the Notch pathway (Lu et al., 2011; Abbassi et al., 2015). For instance, changes in DYRK1A expression (either overexpression or underexpression) stops cell cycle progression at the transition from G0/G1 to S phase, by favoring cyclins D1, D2, and D3 proteasomal degradation (Fernández-Martínez et al., 2015; Hille et al., 2016). Exquisite control of DYRK1A gene dosage is critical, as it can exhibit different (even antagonistic) effects depending on the cellular context and protein kinase expression levels (Fernández-Martínez et al., 2015; Kay et al., 2016). In some cases, opposite levels of DYRK1A expression have similar consequences (Abbassi et al., 2015; Fernández-Martínez et al., 2015). Animal models show that DYRK1A-knockout mice are lethal at the embryonic stage; heterozygous mice are viable but with smaller size, shorter viability and impaired neurological development; and transgenic mice that overexpress DYRK1A show neurodevelopmental delay and cognitive deficits such as hyperactivity, reduced emotionality, altered reference memory, or impaired locomotor development and activity, among others (Fernández-Martínez et al., 2015). However, results obtained in studies with transgenic mice should be interpreted with caution, as the situation may not always represent normal physiological conditions. It is overexpressed in the brains of people with DS, and therefore, it is believed to be involved with the cognitive deficits observed in this syndrome (Fernández-Martínez et al., 2015; Aboudafir, 2017). The activity of DYRK1A protein kinase regulates, by an unknown mechanism, the expression of repressor element 1 silencing transcription factor or neuron-restrictive silencer factor (REST/NRSF) complex (Nižetić and Groet, 2012; Dekker et al., 2014). The function of the REST complex in non-neuronal cells is to silence neuronal genes, whereas in neuronal cells is to regulate pluripotency by inhibiting neuronal differentiation. Proper balance of DYRK1A and REST expression is essential for proper neural development. When DYRK1A is either upregulated or downregulated, it leads to degradation of the REST protein. Conversely, REST can activate DYRK1A transcription. DS animal models show that REST expression is reduced in this syndrome. The imbalance of DYRK1A and REST expression typical of DS results in premature differentiation of neuronal progenitor cells (Canzonetta et al., 2008; Lu et al., 2011; Dekker et al., 2014; Fernández-Martínez et al., 2015; Kay et al., 2016; Wang et al., 2018).

DYRK1A overexpression appears to be also very important in the oncogenesis in people with DS since, when overexpressed, it has a dual effect: on the one hand, it has a tumor-suppressing effect via growth inhibition and premature neuronal differentiation, and, on the other hand, it also has leukemogenic properties, especially in acute myeloblastic leukemia (Créau, 2012; Nižetić and Groet, 2012; Abbassi et al., 2015; Fernández-Martínez et al., 2015; Schneider et al., 2015; Hasle et al., 2016). Conversely, the Notch pathway has a tumor suppressor effect over myeloid malignancies and an oncogenic role in many other tumors. The DYRK1A protein kinase suppresses the Notch signaling pathway, and its reduced action is consistent with the pattern of malignancies typical of DS (Fernández-Martínez et al., 2015; Kay et al., 2016). Moreover, DYRK1A regulates the Sonic Hedgehog (SHH) pathway. Classically, DYRK1A was believed to enhance the SHH activity by increasing Gli1 transcription (Fernández-Martínez et al., 2015; Kay et al., 2016), but paradoxically, this pathway is suppressed in subjects with DS (Wiseman et al., 2009). It was then demonstrated that DYRK1A can also inhibit the SHH pathway by producing a negative regulation of ABLIM proteins, the transcriptional co-activator MKL1 and the actin cytoskeleton (Schneider et al., 2015; Kay et al., 2016). This, together with the typically reduced amount of cerebellar granular neuronal precursors in the cerebellum, could be protecting people with DS from developing medulloblastoma. In addition, the previously referred REST complex is found overexpressed in some medulloblastomas and neuroblastomas to maintain stem cell characteristics. Therefore, the characteristic reduced REST expression in people with DS may also protect them from developing these embryonal tumors (Lu et al., 2011).

However, even though we highlighted the anti-tumoral properties of DYRK1A, it is known to have many other tumorigenic effects depending on the cellular context and the developmental stage. Some examples of DYRK1A oncogenic effects are its antiapoptotic effect or its capacity to phosphorylate STAT3, which is upregulated in a wide range of malignancies (Fernández-Martínez et al., 2015). Actually, STAT3 needs to be phosphorylated by DYRK1A at the S727 phosphorylation site in order to achieve its highest transcriptional activity and, thus, a bigger tumorigenic effect (Fernández-Martínez et al., 2015).

### Tumor Suppressor Genes Present on HSA21

Certainly, it can be assumed that the presence of an extra chromosome in people with DS may confer resistance to the development of tumors by having a greater number of the tumor suppressor genes located in the HSA21. This hypothesis is reinforced by the investigation that demonstrates a reduction in the number of intestinal tumors in mice carrying trisomy for orthologues of HSA21 genes (Ts65Dn or Ts1Rhr mice) and a heterozygous mutation of the APC^Min^ gene, and, on the other hand, an increased incidence of tumors in mice with monosomy of 33 genes orthologues of HSA21 (MsRhr mice) and a heterozygous mutation of the APC^Min^ gene (Sussan et al., 2008; Martel-Billard et al., 2016). The APC^Min^ gene is the murine homolog of the Adenomatous polyposis coli (APC) gene, a key tumor suppressor gene that is frequently inactivated in various intestinal and colorectal cancers, among others.

On HSA21, there are at least four genes described as tumor suppressors (Figure 2). Of those, the ETS2 (V-Ets Avian Erythroblastosis Virus E26 Oncogene Homolog 2) gene (21q22.3) is especially interesting for its capacity to control cell survival through the regulation of multiple targets, such as p53, p21, cyclin D1, preselin-1 and ICAM1, among others (Wolvetang et al., 2003a). Furthermore, when overexpressed, it can have both oncogenic and tumor suppressive roles: it favors the development of acute megakaryoblastic leukemia (Nižetić and Groet, 2012) and prevents development of intestinal tumors (Wolvetang et al., 2003b; Chatterjee et al., 2013).

### Disturbed Metabolism

Metabolism aberrations and oxidative stress are a hallmark both in cancer and in several genetic diseases. *In vitro* and *in vivo* studies show increasing evidence of mitochondrial dysfunction and the presence of a chronic prooxidant state in DS (Pallardó et al., 2010; Izzo et al., 2018). On HSA21, there are at least seven genes involved in mitochondrial function and various enzymes related to detoxification pathways and to lipid, amino acid and carbohydrate metabolism (Aboudafir, 2017; Izzo et al., 2018).

Anatomically, individuals with DS present anomalies in the mitochondria shape and structure, with smaller size, reduced levels of microtubules, and the presence of dense bundles of abnormal filaments. They also exhibit various abnormalities in mitochondrial enzymes, a more rapid loss of the mitochondrial membrane potential, a diminished mitochondrial efficiency and a higher rate of mutations and oxidative damage in the mitochondrial DNA (Pallardó et al., 2010; Perrone et al., 2016; Izzo et al., 2018; Valenti et al., 2018). These alterations have been found across the different types of DS cell. Mitochondrial dysfunction has been associated with the processes of neurodegeneration and aging due to irreversible cell damage (Valenti et al., 2018), but the same mechanism may indeed be responsible for the protection against solid tumors development, at least to some extent.

All these aberrations are tightly related to oxidative stress, which is the imbalance between the production of reactive oxygen species (ROS) and the efficiency of antioxidant defense mechanisms. An excess of ROS has a detrimental effect and is associated with several diseases including, among others, cancer and neurodegeneration. ROS levels have been found to be elevated in neurons, lymphocytes and fibroblasts of people with DS (Martel-Billard et al., 2016). In this regard, the overexpression of the superoxide dismutase 1 or SOD1 gene (21q22.11) present in DS is crucial (Figure 2). Although SOD1 usually has an anti-apoptotic effect, in DS the imbalance between the amount of the cytosolic isoform of SOD1 (increased) and the glutathione peroxidase (normal), induces intracellular accumulation of ROS and, consequently, oxidative stress, cell damage and apoptosis (Hasle, 2001; Ayed et al., 2012; Martel-Billard et al., 2016; Weizmann Institute of Science, 2020).

Similarly, the ETS2 gene (discussed above) can be induced by oxidative stress, thus showing its importance in the control of cell survival (Wolvetang et al., 2003b; Nižetić and Groet, 2012).

### Increased Cellular Apoptosis and Defective DNA Repair System

Remarkably, people with DS have an increased sensitivity to cell apoptosis, and DS cells present a globally increased rate of whole chromosome instability (Nižetić and Groet, 2012; Martel-Billard et al., 2016). This has especially been reported in neurons and lymphocytes (Wolvetang et al., 2003a; Nižetić and Groet, 2012; Aboudafir, 2017). An increased apoptotic response is one of the existing biological barriers to incipient tumor progression. Therefore, cells with DNA damage may be more prone to apoptosis than to malignancy in individuals with DS (Nižetić and Groet, 2012; Hasle et al., 2016).

*In vitro* studies comparing peripheral blood lymphocytes from children with and without DS yield interesting results: DS children have a greater amount of basal endogenous DNA damage compared to healthy controls; when exposed to DNA-damaging agents, this DNA damage is also higher than in non-trisomic controls (in a dose-dependent manner); and when DNA-damaged DS cells are incubated in a repair buffer, these cells not only fail to repair, but DNA damage continues to increase over time (Morawiec et al., 2008).

The tendency to apoptosis together with the defective DNA repair system, could disfavor the production of solid tumors (Hasle et al., 2000).

In addition, telomere length is different not only between euploid and trisomic DS cells but also between different cell tissues in subjects with DS (Nižetić and Groet, 2012). There is evidence of a higher rate of telomere loss in DS people compared with age-matched controls, which may be linked to the higher rate of hematologic malignancies and neurogeneration (Sukenik-Halevy et al., 2011). This tendency could be secondary, at least in part, to the increased chromosomal instability induced by critically short telomeres (Calado et al., 2012).

Among the genes of HSA21 related to apoptosis and response to DNA damage are to be highlighted the aforementioned DYRK1A and ETS2 genes (Figure 2).

DYRK1A, in addition to its previously referred role in neurodevelopment, is also closely related to apoptosis and cell cycle regulation. It has the ability to both stimulate and inhibit apoptosis in different developing tissues by regulating the activity of p53, Notch and caspase 9 (Nižetić and Groet, 2012). DYRK1A has been linked to the DNA damage response in two ways: firstly, by phosphorylating (and activating) sirtuin-1 (SIRT1), which leads to p53 deacetylation and thus to an inhibition of apoptosis despite the presence of genotoxic stress (Fernández-Martínez et al., 2015; Kay et al., 2016); and secondly, by its interaction with the E3 ubiquitin ligase RNF169 (Roewenstrunk et al., 2019). In response to DNA damage, there is a negative feedback loop between DYRK1A and p53: DYRK1A phosphorylates (and activates) p53, which subsequently induces the expression of miR1246 that, in turn, downregulates DYRK1A expression (Zhang et al., 2011; Nižetić and Groet, 2012). Even though DYRK1A is mainly considered an antiapoptotic gene through the phosphorylation (and inactivation) of the proapoptotic caspase 9 (Fernández-Martínez et al., 2015), it can also have a proapoptotic effect in some contexts in a dosage-dependent manner (Kay et al., 2016).

In the same way, the ETS2 gene (also discussed above) is related to apoptosis through different targets that include p53, bcl-xL and p21 (Wolvetang et al., 2003b; Nižetić and Groet, 2012; Chatterjee et al., 2013). When overexpressed, it increases sensitivity to apoptosis through p53 signaling pathway, as long as the downstream factors of the pathway remain unaltered (Wolvetang et al., 2003b; Nižetić and Groet, 2012). Studies using transgenic mice show that its overexpression leads to increased neuron apoptosis through activation of caspase 3 (Wolvetang et al., 2003a).

### Angiogenesis

People with DS have an overexpression of antiangiogenic factors and a lower incidence of angiogenesis-related diseases (e.g., diabetic retinopathy, atherosclerosis). Likewise, this antiangiogenic environment may be responsible, at least to some extent, for the lower incidence of certain solid tumors (Baek et al., 2009; Kay et al., 2016). This mechanism would support the fact that some tumors that develop independently from angiogenesis (e.g., leukemia) are not at a reduced incidence and those who directly depend on angiogenesis are less frequent. But conversely, some tumors with an elevated angiogenesis are not uncommon (e.g., testicular tumors) (Nižetić and Groet, 2012). It should be noted that a diminished angiogenesis would be especially important in halting tumor progression, but of lesser importance in preventing its occurrence (Ryeom et al., 2009; Nižetić and Groet, 2012). This concept correlates with the fact that individuals with DS have frequent benign or indolent neoplasms but significantly less amount of aggressive tumors (Ryeom et al., 2009). Murine models of DS (e.g., Ts65Dn, Tc1) show reduced angiogenesis and thus, reduced tumor growth (Reynolds et al., 2010; Ayed et al., 2012). This decrease in angiogenesis is especially important in xenografts of very aggressive tumor cell lines, but to a lesser extent in endogenous tumors of murine models or in xenografts of newly derived cell lines (Nižetić and Groet, 2012). Genes involved in decreased angiogenesis in individuals with DS include Col18A1, RCAN1, and DYRK1A (Figure 2).

The Col18A1 (Collagen Type XVIII Alpha 1 Chain) gene (21q22.3) encodes the alpha chain of collagen XVIII, which is transformed after proteolysis into endostatin. Endostatin is a potent inhibitor of angiogenesis through inhibition of the pro-angiogenic factor VEGF-A (vascular endothelial growth factor A), and it is also related to migration and proliferation (Weizmann Institute of Science, 2020). Endostatin is increased in people with DS (Ayed et al., 2012; Nižetić and Groet, 2012; Martel-Billard et al., 2016). Murine models deficient in endostatin have an enhanced angiogenesis and an increase in tumor growth, and conversely, murine models with a 1.6-fold increase in endostatin levels (resembling DS condition) have the opposite effect: angiogenesis and tumor growth are suppressed (Sund et al., 2005).

The RCAN1 (regulator of calcineurin 1) gene (21q22.12), previously known as DSCR1, negatively regulates calcineurin and decreases the expression of VEGF-A through the VEGF-calcineurin-NFAT (Nuclear Factor of Activated T-cells) signaling pathway (Baek et al., 2009; Nižetić and Groet, 2012; Martel-Billard et al., 2016). In individuals with DS, RCAN1 is overexpressed (Baek et al., 2009), and, consistently, the NFAT signaling pathway is inhibited (Birger and Izraeli, 2012). The NFAT pathway inhibition is very important in the pathogenesis of this syndrome, by regulating the immune response in leukocytes, bone homeostasis, cardiac development and tumor progression (Suehiro et al., 2014). This pathway is considered oncogenic by promoting angiogenesis, metastatic dissemination and lymphoid proliferation (Birger and Izraeli, 2012; Fernández-Martínez et al., 2015). Ts65Dn mice inoculated with either B16F10 melanoma or Lewis lung carcinoma cells exhibit suppression of tumor growth compared to littermate controls, secondary to decreased angiogenesis (Baek et al., 2009). This antiangiogenic effect seems to be closely related to the extra RCAN1 gene. The rational for this hypothesis is that the antiangiogenic effect is maintained in transgenic mice with only one extra copy of the RCAN1 gene (instead of the 104 trisomic genes of the Ts65Dn mouse), and still is much lower compared to the murine model with two copies of RCAN1 and three copies of the remaining 103 genes (Baek et al., 2009). However, RCAN1 cannot be the only gene responsible for the antiangiogenic properties of individuals with DS, since such diminished angiogenesis has also been found in the Tc1 mouse model (that has only two copies of RCAN1) (Reynolds et al., 2010; Ayed et al., 2012).

Overexpressed DYRK1A (previously referred to) also attenuates the NFAT signaling pathway (Fernández-Martínez et al., 2015). This attenuation by DYRK1A overexpression is believed to have a tumor suppressive effect in solid tumors but, conversely, promote the development of some lymphoproliferative disorders, e.g., megakaryocytic malignancies in children with DS (Birger and Izraeli, 2012; Nižetić and Groet, 2012; Fernández-Martínez et al., 2015; Kay et al., 2016). This makes DYRK1A a key gene in the unique profile of tumors in individuals with DS. However, its protective effect is controversial since, as mentioned above, it can also inhibit apoptosis and this could favor tumor cell survival. In general population tumors, the expression of DYRK1A is controversial; for example, it is underexpressed in melanoma and overexpressed in metastatic pancreatic cancer and cervical cancer caused by human papillomavirus 16 (Weizmann Institute of Science, 2020).

### Protective Effect of the Tumor Stroma

The tumoral microenvironment or stroma has an important role in the process of tumorigenesis, during which it is variable and dynamic. Solid tumors are not just a conglomerate of tumor cells, but also contain many other different substances and cell types that form the extracellular matrix and, collectively, promote tumor progression.

The extracellular matrix and fibroblasts of people with DS do not seem to be permissive to promote the proliferation and survival of tumor cells (DownCiclopedia, 2019; Yu et al., 2020). In subjects with DS, these fibroblasts and extracellular matrix have particular characteristics, with differences in types I, II, III, V and VI collagens, matrix metalloproteinase 2 and hyaluronan (Bénard et al., 2005; Satgé and Bénard, 2008; Satgé et al., 2008). Fibroblasts of people with DS have a reduced capacity of migration and proliferation (Nižetić and Groet, 2012).

Research in this field is still scarce. *In vitro* studies show that the extracellular matrix secreted by fibroblasts from individuals with DS suppresses the proliferation of the breast cancer cell line MDA-MB-431 compared to the matrix produced by control fibroblasts (Bénard et al., 2005; Satgé and Bénard, 2008; Satgé et al., 2008). Contrarily, there was no growth inhibition when the same breast cancer cell line was co-cultured neither *in vitro* with only DS fibroblasts nor in xenografts in nude mice. Other studies using extracellular matrix secreted by trisomy 21 fibroblasts revealed a reduction in neuroblastoma cell density when non-hyperaggressive neuroblastoma cell lines (i.e., SK-N-SH and SK-N-As) were used, but not when the highly MYC-amplified IGR-N-91 neuroblastoma cell line was tested (Satgé et al., 2008).

These findings suggest that the characteristic microenvironment of individuals with DS as a whole, but not their fibroblasts alone, is not conducive to promoting tumor cell proliferation and survival (Ayed et al., 2012). This would provide an explanation for people with DS having a lower incidence of stromal-rich tumors (e.g., breast cancer) but not stromal-poor tumors (e.g., lymphoproliferative disorders, retinoblastoma and germ cell tumors) (Bénard et al., 2005; Satgé and Bénard, 2008; Satgé et al., 2008; Martel-Billard et al., 2016).

### Impaired Immune System Response

The immune system has a pivotal role in tumorigenesis. Individuals with DS are characteristically immunosuppressed, with increased susceptibility, severity and mortality from infections, and at increased risk of developing autoimmune diseases. The anomalies concerning the immune system and lymphoid series in DS are varied and are present at a very early stage of development. They present thymic hypoplasia and hypofunction, B and T cell lymphopenia, an inverted CD4/CD8 T cell ratio (with increased proportion of CD8^+^ and reduction of CD4^+^ T lymphocytes), impaired function of neutrophils, B, T, and NK cells, post-thymic lymphocyte maturation defects, chemotactic and phagocytic abnormalities, aberrations in humoral immunity and altered levels of total immunoglobulins and their subclasses (Nižetić and Groet, 2012; Satgé and Seidel, 2018; Araya et al., 2019). People with DS present a chronic inflammatory state and, as previously referred, their lymphocytes show an increased rate of apoptosis, which could explain their lymphopenia (Nižetić and Groet, 2012; Araya et al., 2019).

There are numerous genes located on HSA21 that could be responsible for this condition (Figure 2). In fact, it seems that oxidative stress secondary to the imbalance between SOD1 and GPX/catalase would also contribute to the altered immune response of individuals with DS, especially in relation to reduced neutrophil action and impaired phagocytosis (Muchová et al., 2014). Moreover, the NFAT signaling pathway aberration secondary to the action of the previously referred RCAN and DYRK1A genes also participates in the modulation of the immune response (Satgé and Seidel, 2018). Regarding autoimmunity, subjects with DS are found to have increased levels of cytokines related to it. Of those, the interferon seems to be one of great importance, since four out of six interferon receptor units are encoded by HSA21, and transcriptomics analysis show a constant hyperactive interferon signaling (Araya et al., 2019).

Such immune dysfunction should predictably result in an increased incidence of cancers, but this does not occur (Nižetić and Groet, 2012; Satgé and Seidel, 2018). The reason has not yet been clarified. Some authors postulate that it could be secondary to the characteristic increase in the proportion of gamma/delta T cells in DS, which are responsible for tumor immunosurveillance, favoring early inhibition of tumor growth (Nižetić and Groet, 2012; Yu et al., 2020). More research is definitely needed in this area.

### Epigenetics

Epigenetic alterations in DS affect not only HSA21 but also the entire genome. Subjects with DS have an aberrant pattern of methylation, with some areas of hypermethylation and others with hypomethylation across the entire genome (including genes outside HSA21) (Antonarakis et al., 2020; Yu et al., 2020; Dekker et al., 2014; Weizmann Institute of Science, 2020). Studies over the past 10 years show that people with DS have epigenetic changes on HSA21 that are recurrent and reproducible in different tissues and cell subtypes (Yu et al., 2020). The aberrant methylation pattern occurs in early development and has especially been proved in brain, placenta and lymphocytes (since they correspond to the most investigated features of DS) (Kerkel et al., 2010; Dekker et al., 2014; Yu et al., 2020). In addition, people with DS and their mothers have altered folate metabolism, which is strongly involved in methylation processes (Hobbs et al., 2000; Rabin and Whitlock, 2009; Aboudafir, 2017). Furthermore, within HSA21 there are some genes that can produce post-translational histone modifications and thus contribute to the epigenetic aberrations in DS. Histone modifications are thought to be related to the presence of mitochondrial dysfunction in DS, but this field requires further investigation (Dekker et al., 2014). Genes related to the epigenetic landscape include the above-mentioned SOD1, DYRK1A and ETS2, and other genes such as DNMT3L and CBS, among others (Figure 2; Dekker et al., 2014; Antonarakis et al., 2020; Yu et al., 2020).

The DNMT3L (DNA Methyltransferase 3 Like) gene (21q22.3) encodes for a protein with methyltransferase-like activity (favoring methylation) and with the ability to suppress transcription through its interaction with histone deacetylase 1. It also plays a crucial role in the embryonic progenitor cells, acting both as a positive and negative regulator of DNA methylation. Its overexpression could account, at least to some extent, for the methylation abnormalities of DS (Dekker et al., 2014; Weizmann Institute of Science, 2020; Yu et al., 2020).

The CBS (cystathionine beta-synthase) gene (21q22.3), encodes the CBS protein, which is found overexpressed in DS. This overexpression results in decreased amounts of homocysteine and methionine (due to substrate depletion), which may affect DNA methylation at both nuclear and mitochondrial DNA levels (Dekker et al., 2014; Aboudafir, 2017; Antonarakis et al., 2020; Yu et al., 2020; Zuhra et al., 2020).

All these epigenetic aberrations could be of great importance. Unlike adult tumors, tumors of childhood are characterized by low mutational burden and few known risk factors. In this sense, epigenetics is progressively gaining prominence in the pathophysiology of pediatric solid tumors, specifically in embryonal tumors (Lawlor and Thiele, 2012). This, together with its characteristic genome-wide effect, may be part of the reason why children with DS do not develop some pediatric tumors.

### Global Effects on Other Genes Beyond HSA21

All these mechanisms become even more complex when taking a global view of the individual. A very interesting study (Letourneau et al., 2014), analyzed the mRNAs present in the fibroblasts of two homozygous twins, one with DS and one without. In this study, differences in expression between the twins were evident in 182 genes, located not only on HSA21 but also throughout the entire genome. In addition, it was found that the difference in gene expression between disomic and trisomic cells was organized into chromosomal regions that the authors called GEDD (gene expression deregulation domains), which were maintained when the fibroblasts were transformed into pluripotent stem cells.

Similarly, transcriptomic analyses show that the extra HSA21 is accompanied by changes in the expression of gene products located throughout the genome. This global disturbance in the genome behavior increases the difficulty in establishing genotype-phenotype relationships (Hasle, 2001; Hasle et al., 2016; DownCiclopedia, 2019; Yu et al., 2020).

In terms of global actions, the increasingly well-known microRNAs (miRNAs) are of great importance, since they are capable of acting on multiple targets. Thus, a single miRNA can regulate several biological processes (Bras et al., 2017). To date, HSA21 is known to contain up to 30 miRNAs (Griffiths-Jones, 2004; miRBase, 2004); of those, five are overexpressed and related to the DS phenotype: let-7c, miR-99a, miR-125b2, miR-155 and miR-802 (Figure 2; Bras et al., 2017). Their overexpression implies decreased expression of the proteins encoded by the mRNA targeted by these miRNAs (Nižetić and Groet, 2012; Weizmann Institute of Science, 2020). If some of these proteins were oncogenic, this would protect individuals with DS from developing tumors. In fact, more than 3,630 genes are potentially regulated by these five miRNAs, including many of the genes and pathways discussed above (Zhao et al., 2017). Hence, overexpression of miRNAs could explain, at least partially, the genome-wide impact of the additional HSA21.

Let-7c, which clusters with miR99a and miR125b2 on HSA21, is involved in neurodevelopment and, when overexpressed, impairs neuronal development and functionality (McGowan et al., 2018). It has been found at low levels in prostate cancer, some lung tumors, some breast malignancies and in Wilms tumor (Fores-Martos et al., 2015; Bras et al., 2017), which correlates with the low incidence of these tumors in DS.

MiR99a exerts a negative regulation on the STAT cascade and on the signaling pathway mediated by interleukin-6. It is associated with DS, diabetes mellitus and endometriosis (Weizmann Institute of Science, 2020). Regarding cancer, it is related to ovarian serous carcinoma, acute myeloid leukemia and diffuse large B-cell lymphoma; it is downregulated in lung cancer, breast cancer and melanoma; and inhibits the proliferation of prostate cancer cells (Fores-Martos et al., 2015; Bras et al., 2017; Zhao et al., 2017).

MiR125b2, a paralog of miR125b1 (Wang et al., 2019), has an important role in early hematopoiesis, inflammation, immune system development, host immune defense and the occurrence of autoimmune diseases (Sun et al., 2013). It is especially important in DS because it can act both as a tumor suppressor and oncomiR (Sun et al., 2013). It has been reported to act as a tumor suppressor by inhibiting migration and inducing senescence or apoptosis in a long list of malignancies that include, among others, prostate cancer, some breast cancers, some lung cancers, hepatocellular carcinoma (Sun et al., 2013; Bras et al., 2017; Wang et al., 2019). However, it is most frequently considered an oncomiR by avoiding apoptosis and favoring migration and metastasis in many other malignancies, such as leukemia, lymphoma, colorectal cancer and some breast and lung cancers, among others (Sun et al., 2013; Wang et al., 2019). It has many important direct targets (e.g., p53, Bcl2, Bak1, Bright/ARID3a, E2F2, IRF4, and MYC) and is related to different signaling pathways (mainly NFkb, TGFb, Wnt, PI3K and SHH) (Wang et al., 2018; Wang et al., 2019), which explains its capacity to produce such variable effects.

MiR155 regulates both innate and adaptative immune responses and is a powerful pro-inflammatory factor. It is a key player in the process of oncogenesis, acting both as a tumor-suppressor miRNA and oncomiR depending on the cell type, the stage of evolution and the miR155 levels of expression. This way, in some malignancies intermediate miR155 levels have oncogenic properties but, in others, high miR155 levels have antiproliferative actions and enhance anti-tumor activity of NK cells (Michaille et al., 2019). This suggests that overexpression of miR155 in DS has an anti-tumor effect. MiR155 is related to various important signaling pathways, e.g., NFkb and PI3K, and has important direct targets such as MeCP2, a protein involved in the processes of neuron formation and development, known to be downregulated in DS (Bras et al., 2017; Michaille et al., 2019). Moreover, miR155 is also a key regulator of mitochondrial activity and biogenesis (Valenti et al., 2018), which are highly relevant processes in the pathophysiology of DS and cancer.

MiR802 regulates over 650 genes and is related to various diseases such as cancer (e.g., leukemia, malignant breast, prostate and pancreatic tumors, among others), obesity, diabetes mellitus, etc. Its overexpression also contributes to the reduced levels of MeCP2 (Bras et al., 2017; Zhao et al., 2017).

### Non-Genetic Factors

Apart from the purely genetic part, it is important to take into account other factors that are inherent to the lifestyle of people with DS and that protect or expose them to the development of malignancies. In one sense, they often have less exposure to known carcinogens (alcohol, tobacco, hormone treatments, ultraviolet radiation, less sexual activity and fewer occupational carcinogens, among others) (Bratman et al., 2014); but conversely, they have other risk factors that are more prevalent than in the general population (e.g., obesity, sedentary lifestyle and nulliparity). The former may justify only partially the difference in the tumor incidence, since other tumors involving the same carcinogens do not have a decreased incidence in DS (Satgé et al., 2013). Overall, the intrinsic protective effect of trisomy 21 ought to compensate for the risk factors of subjects with DS, given that the incidence of many of these tumors is not increased (Hasle, 2001; Satgé et al., 2013; Hasle et al., 2016; Martel-Billard et al., 2016).

Many authors postulate that protection in the development of solid tumors is closely related to the shorter average life expectancy of individuals with SD, currently around 60 years of age (Antonarakis et al., 2020; Rethoré et al., 2020). In contrast, several studies show that the age-adjusted incidence of cancer remains much lower than expected (Hasle et al., 2000; Patja et al., 2006; Hasle et al., 2016; Jørgensen et al., 2019). Nonetheless, by focusing on tumors of pediatric age, this and other biases (e.g., lifestyle-related) can be eliminated, so that the results obtained should be directly related to the presence of an additional HSA21.

## Conclusion and Outlook

After this extensive review, it is clear that the absence of most solid tumors in individuals with DS is not secondary to a few mechanisms. Most likely, there are multiple factors contributing to this unique situation and some of them may even have a synergistic effect. It is also evident that more experimental research is required to finally elucidate the underlying molecular mechanisms that protect these people against developing these tumors. These ten reasons establish the starting points from which research needs to be continued. From here, we can have a clarified vision of the objectives of the research and the paths to follow.

Increasing our knowledge about the causes that disfavor the development of these tumors will help us enhance our understanding of the pathogenesis of certain tumors and therefore the development of new advanced therapies and prevention programs.

Additionally, by focusing our research on the positive aspects of DS, we will enhance further research on this syndrome. To date, many initiatives and projects on DS have been developed with positive results, which have enabled people with DS to acquire physical and neurological skills that allow them to have a much more normal life nowadays. Despite this, research on DS is still very much needed. By this review, we highlight the importance of not abandoning the research in DS.
